# Abdominal Pain: A Comparison between Neurogenic Bowel Dysfunction and Chronic Idiopathic Constipation

**DOI:** 10.1155/2013/365037

**Published:** 2013-09-15

**Authors:** Pia Møller Faaborg, Nanna Brix Finnerup, Peter Christensen, Klaus Krogh

**Affiliations:** ^1^Neurogastroenterology Unit, Department of Hepatology and Gastroenterology, Aarhus University Hospital, Dk-8000 Aarhus, Denmark; ^2^Pelvic Floor Unit, Department of Surgery P, Aarhus University Hospital, Dk-8000 Aarhus, Denmark; ^3^Danish Pain Research Centre, Aarhus University Hospital, Dk-8000 Aarhus, Denmark

## Abstract

*Introduction*. Most spinal-cord-injured patients have constipation. One-third develop chronic abdominal pain 10 years or more after injury. Nevertheless, very little is known about the nature of abdominal pain after spinal cord injury (SCI). It may be neuropathic or caused by constipation. *Aim*. To compare characteristics of abdominal pain in SCI with able-bodied with chronic idiopathic constipation (CIC). *Subjects and Methods*. 21 SCI and 15 CIC patients were referred for treatment of bowel symptoms. Constipation-related symptoms were assessed with the Cleveland Constipation Scoring System and the International Spinal Cord Injury Basic Bowel Function Data Set. Characteristics of abdominal pain were described using the Brief Danish Pain Questionnaire. Total gastrointestinal transit times (GITT) were measured by radiopaque markers. *Results*. Seventeen (81%) SCI and 14 (93%) CIC patients reported abdominal pain or discomfort within the last month (*P* = 0.38). Pain was considered more intense by CIC than by SCI patients (*P* < 0.05). Only minor differences were found in patient's qualitative description of abdominal pain or in the location of pain. In neither SCI nor CIC was pain associated with GITT. *Conclusion*. Most characteristics of abdominal pain among SCI patients resemble those of CIC. This indicates that constipation is a major cause of pain after SCI.

## 1. Introduction

Spinal cord injury (SCI) has severe consequences for colorectal and anal sphincter function. The term neurogenic bowel dysfunction (NBD) has been introduced and includes constipation, faecal incontinence, and abdominal pain [1]. It is well documented that faecal incontinence affects up to 75% and constipation approximately 80% of subjects with SCI [2]. In contrast to other symptoms, abdominal pain after SCI has received very little attention. Abdominal pain can have severe consequences for the quality of life of SCI patients [3]. It usually has a late onset as it is present in only 5–10% after five years [4] but in one-third after 10 years or more [5].

 The nature of abdominal pain in NBD is unknown. This is unfortunate as the choice of treatment should reflect the underlying cause. We have previously described an association between infrequent defecation and abdominal pain suggesting a relation to constipation [5]. This is consistent with the fact that most able-bodied patients with chronic constipation have abdominal pain [6]. Abdominal pain in SCI is sometimes considered neuropathic pain if no underlying visceral pathology is identified. Neuropathic pain is present in other parts of the body in about 50% of SCI patients [4]. In general, at-level neuropathic pain has an earlier onset than below-level neuropathic pain, but both types typically have an onset earlier than reported for abdominal pain [4, 7]. This argues against neuropathic pain as a cause of abdominal pain. Nevertheless, it remains to be shown whether neuropathic pain affects the gut. Diagnosing neuropathic abdominal pain using the standard grading system is difficult as it involves sensory testing in the pain area [8]. In addition, pain due to constipation may be described differently in the spinal-cord-injured patients because of the central nervous system damage.

 We hypothesised that abdominal pain in NBD is both neuropathic and visceral. Accordingly, we further hypothesised that characteristics of abdominal pain in NBD are different from those of chronic idiopathic constipation (CIC) and only weakly associated with prolonged gastrointestinal transit time. 

 The aims of the present study were threefold: to describe the characteristics of abdominal pain in patients with SCI, to compare the characteristics of colorectal function and abdominal pain in patients with SCI and patients with CIC, and, finally, to investigate the association between abdominal pain and gastrointestinal transit time in patients with SCI.

## 2. Materials and Methods

Between September 2009 and February 2012, 21 adult SCI patients with NBD and 15 able-bodied patients with CIC were consecutively included from the Anorectal Physiology Unit at Aarhus University Hospital, Denmark. International standards for the classification of spinal cord injury [9] and the autonomic standard assessment form [10] were used for description of background characteristics of SCI patients. All patients with CIC fulfilled the Rome III criteria for this diagnosis [11]. Exclusion criteria were other organic bowel disease, previous major intra-abdominal surgery, major psychiatric disease, and inability to give informed consent. The study was conducted according to Helsinki Declaration II and approved by the Danish Data Protection Agency (number 2009-41-3982).

### 2.1. Assessment of Bowel Function and Abdominal Pain

#### 2.1.1. Bowel Function

Constipation was assessed with the Cleveland Constipation Scoring System [12] consisting of 8 questions with a score ranging from 0 to 30. In subjects with SCI further information was obtained through the International Spinal Cord Injury Basic Bowel Function Data Set [13]. 

### 2.2. Abdominal Pain and Discomfort

Both patient groups filled in a questionnaire composed of 18 questions describing pain or discomfort in the abdomen within the last month. Items included were location of pain/discomfort on a body chart, pain descriptors from a Brief Danish Pain Questionnaire (list of 18 descriptors) [14], temporal aspects, and whether the pain/discomfort was accompanied with nausea, sweating, abdominal muscle tightness, or sensitive skin for touch. Intensity of average pain, unpleasantness, and maximum pain within the last week was rated on a numeric rating scale (NRS 0–10, with 0 indication no pain/unpleasantness and 10 worst imaginable pain/unpleasantness), and patients rated the impact of pain/unpleasantness on daily activities, mood, and sleep also on a 0–10 NRS. Furthermore, patients were provided with a list of possible alleviating and aggravating factors and asked for current treatment of abdominal pain.

### 2.3. Assessment of Gastrointestinal Transit Time

Total gastrointestinal transit time (GITT) was determined as part of the standard clinical evaluation of patients and performed as described by Abrahamsson et al. [15], At 12 a.m. for six consecutive days, subjects ingested a capsule containing 10 radiopaque markers, and a plain radiography of the abdomen was taken on the seventh day. The number of markers left in the colorectum was counted, and the GITT was calculated based on the following formula:

(1) $$\begin{array}{c} \text{GITT}=\frac{\left(M+\left(f\times D\right)\right)}{D}, \end{array}$$

where *M* is the total number of markers left, *D* is the number of markers ingested each day, and *f* is the fraction of the daily markers selected for the provision of transit. In this case *f* = 0.5.

### 2.4. Statistical Analysis

Differences between the two patient groups were compared using Fisher's exact test or Mann-Whitney *U* test, whenever appropriate. Correlation between nonparametric variables was assessed using Spearman's correlation. *P* < 0.05 was considered statistically significant.

## 3. Results

Clinical and basic characteristics of patients are shown in Table 1. In general, patients with SCI were older and included more males than those with CIC (Table 1). General information about autonomic dysfunction is included in Table 1.

### 3.1. Colorectal Function

The Cleveland Constipation Score was significantly higher in patients with CIC (16, 9–25) (median, range) than in patients with SCI (11, 4–16) (median, range) (*P* = 0.01). Furthermore, the clinical presentation of constipation was fundamentally different in the two groups (Table 2). Patients with SCI usually had no (38%) or indirect (29%) awareness for defecation, and 71% relied on anorectal stimulation either digitally or with suppositories or enema. This was usually painless and ensured frequent bowel movements. In contrast, patients with CIC had normal awareness of defecation and relied on oral laxatives. They avoided anorectal manoeuvres for evacuation, which was infrequent and often difficult or painful. 

 Haemorrhoids and perianal sores were reported by 1 (5%) and 5 (24%) of SCI patients. Six (29%) had incontinence more than once a week, and two (10%) had daily faecal incontinence using a pad all the time. No patients with CIC had perianal disease or faecal incontinence. 

### 3.2. Characteristics of Abdominal Pain and Discomfort

Seventeen out of 21 (81%) with SCI and 14 out of 15 (93%) patients with CIC reported abdominal pain or discomfort within the last month (*P* = 0.38). Pain was, however, considered more intense and unpleasant by those with CIC (Table 3). Pain usually lasted less than one hour in six patients with SCI and five with CIC, between 1 and 24 hours in seven and five patients, and more than 24 hours in three and four patients, respectively. There was no significant difference in accompanying symptoms as 53% of SCI and 79% of CIC patients reported nausea, 41% and 36% reported sweating, 47% and 57% reported muscle tightness, and 29% and 7% reported increased skin sensitivity (all *P* > 0.19, Fisher's exact test). Further characteristics are presented in Table 3.

**Figure 1:**
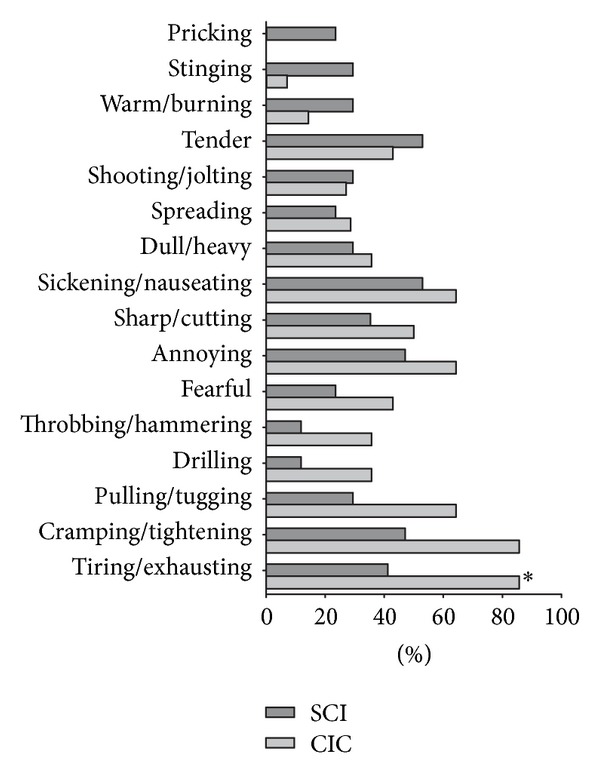
The Brief Danish Pain Questionnaire [14].

The qualitative description of pain as assessed with the Brief Danish Pain Questionnaire is presented in Figure 1. Although the only statistically significant difference was that term “tiring/exhausting” was used more often by the CIC group, SCI patients tended more often to report descriptors such as “pricking,” “stinging,” and “warm/burning.” 

 The location of pain was similar in the two groups. Thus, two patients with SCI and two with CIC had upper abdominal pain, nine with SCI and seven with CIC had lower abdominal pain and four with SCI versus five with CIC had periumbilical pain. Six subjects (five with SCI and one with CIC) did not fill in the body chart.

### 3.3. Gastrointestinal Transit Time versus Pain and Constipation Score

There was no association between total GITT and average intensity of pain or unpleasantness in neither patients with SCI (*P* = 0.95, *r* = 0.015 and *P* = 0.53, *r* = −0.15) nor those with CIC (*P* = 0.55, *r* = 0.17 and *P* = 0.57, *r* = −.16). Likewise, there was no association between the Cleveland Constipation Score and total GITT in neither SCI (*P* = 0.13, *r* = 0.35) nor in CIC patients (*P* = 0.18, *r* = 0.37). 

### 3.4. Level of Spinal Cord Injury versus Pain and Constipation Score

The SCI group were subdivided into two: high SCI, *n* = 14 (cervical and thoracic SCI), and low SCI, *n* = 7 (lumbar SCI). There was no association between neurological level of SCI and abdominal pain or discomfort as 11 of 14 (79%) with high SCI and 6 of 7 (86%) with low SCI had pain, *P* = 0.70. Likewise, there was no association between neurological level of SCI and the Cleveland Constipation Score as the high SCI group had a median constipation score of 11 (range 4–16) and the low SCI group had a median constipation score of 11 (range 4–16) (*P* = 91).

## 4. Discussion

In the present study abdominal pain and discomfort were highly prevalent in subjects with SCI and NBD as well as those with CIC. In both groups pain had significant impact on daily functions, mood, and, in patients with CIC, also sleep. Abdominal pain in CIC is common and well described in the literature. Based on the present data, we find that abdominal pain is important among patients with NBD too. Even though many similarities were found between abdominal pain in CIC and NBD, there were differences. Specifically, abdominal pain was considered significantly more intense and unpleasant by patients with CIC, and, accordingly, it affected their mood and sleep more. We can only speculate to what degree these differences reflect different referral patterns of the two patient groups for treatment at our unit, reduced visceral sensation in SCI, or real differences in the underlying pathophysiology.

 Neuropathic pain in SCI is often described by patients as “pricking,” “stinging,” “warm,” and “burning” [7, 16]. In the present study these descriptors were used more often about abdominal pain by patients with SCI than by those with CIC. The differences did not reach statistical significance, which may be a type II error due to the relatively low number of patients in each group. Although not statistically significant, SCI patients more often reported that abdominal pain was associated with sensitive skin. These results may suggest that neuropathic pain explains some cases of abdominal pain in SCI. However, it cannot be excluded that patients with SCI are unable to separate their abdominal pain from a concurrent neuropathic pain or that SCI patients describe visceral pain differently because of their nervous system lesion. For other descriptors of pain there was a very large overlap between SCI and CIC, and in both groups food intake increased abdominal pain or discomfort in about half of patients. This is consistent with the hypothesis that abdominal pain in SCI is mainly caused by constipation. This is also consistent with previous studies showing that abdominal pain usually has a late onset after SCI [3, 5, 17] and that the severity of constipation is significantly associated with time since injury [1]. Furthermore, neuropathic pain in other parts of the body usually has an earlier onset than abdominal pain in SCI [3, 4]. Dividing SCI patients into those with a high lesion and those with low SCI further adds to the thesis that abdominal pain is mainly caused by constipation as no association between level of SCI and abdominal pain was found. However, numbers are small to draw any firm conclusions. 

 Speaking against constipation as the main cause of pain in NBD, we found no association between the severity of abdominal pain and GITT assessed by radiopaque markers. This may not be surprising, as no association has been found between GITT and other symptoms of NBD [18]. Likewise, we found no association between abdominal pain and GITT among the able-bodied patients with CIC. GITT mainly reflects colorectal transit time, as the passage through the colon is much slower than through the small intestine. We have previously shown that patients with SCI not only have prolonged colorectal transit, but also significantly prolonged small intestinal transit [19]. To what degree slow small intestinal transit contributes to abdominal pain in NBD remains to be shown. 

 The pathophysiology of constipation in NBD is poorly understood. Patients with conal or cauda equina lesions have reduced tone and reflex activity of the distal colorectum [20, 21]. This impairs evacuation of stools [22] and causes prolonged transit of the descending colon and the rectosigmoid [23]. Patients with supraconal lesions have increased colorectal tone and reflex activity. This is associated with prolonged transit throughout the colorectum [23–28]. Even though there are minor differences in the clinical presentation of constipation in subjects with supraconal and conal/cauda equina lesions, our data illustrate how constipation in NBD is very different from that in CIC. Lack of normal sensation for defecation and the risk of faecal incontinence make many NBD patients rely on digital anorectal stimulation, suppositories, and enema. Induced bowel evacuation may be time consuming and cause perianal problems, but in most SCI patients it is not associated with pain or severe discomfort. In contrast, our patients with CIC usually took oral laxatives and very few used digital stimulation or enema. This is consistent with results from a previous study of transanal irrigation in various groups of patients. Those with CIC had significantly less benefit from irrigation than those with NBD [29]. 

 In light of the fundamental differences between NBD and CIC the choice of methods for evaluation of bowel function is questionable. We choose the Cleveland Constipation Scoring System, often termed the Wexner Constipation Score [12]. The score is very commonly used, but its validity in NBD has not been tested. In contrast, the International Spinal Cord Injury Basic Bowel Function Data Set [13] is valid in subjects with SCI, but its use is restricted to this group. No symptom-based scoring system for neuropathic visceral pain exists. Therefore, we relied upon the Brief Danish Pain Questionnaire. An alternative would have been applying various standardized mechanical, thermal, electrical, or chemical stimuli to the colorectal wall and registering the sensation felt by the patients. A method for this has recently been described and could be used in future studies [30].

 Selection of patients may have had significant effects on the results of the present study. Patients with SCI are referred to our unit for various reasons, mainly including faecal incontinence and constipation. Therefore, constipation needed not be the main complaint of all SCI patients. In contrast, most patients with CIC are treated in primary care or in regional hospitals. Only a minority with severe symptoms are referred for evaluation at our unit. These would probably include those with most severe pain.

 Based on the present study we conclude that, even though abdominal pain in some patients with SCI has characteristics of neuropathic pain, most characteristics resemble those reported by patients with CIC. Therefore, we find it likely that constipation is a major cause of abdominal pain in NBD. However, intervention studies including assessment of abdominal pain in NBD patients successfully treated for constipation and studies of abdominal pain and small intestinal dysmotility in NBD should be performed before firm conclusions can be made.

## Figures and Tables

**Table 1:** Patient characteristics.

	Spinal cord injury	Chronic idiopathic constipation	*P* value
Number	21	15	
Gender (female/male)	9/12	13/2	0.014
Age, years, mean (SD)	45.2 (14.2)	32.3 (11.7)	0.007
Abdominal pain or discomfort^a^, *n* (%)	17 (81.0)	14 (93.3)	0.38
Neurological level^b^			
Cervical, *n* (AIS^b^, *n*)	4 (A 3, C 1)		
Thoracic (Th3-10), *n* (AIS^b^, *n*)	10 (A 7, C 3)		
Lumbar, *n* (AIS^b^, *n*)	7 (A 1, C 5, D 1)		
Completeness			
Complete, *n *	11		
Incomplete, *n *	10		
Abnormal control^c^			
Blood pressure	5*		
Heart rate	5*		
Sweating	5*		
Normal control^c^			
Temperature	21		
Bronchopulmonary system	21		

^a^Within the last month.

^b^International standards for the classification of spinal cord injury.

^c^The autonomic standard assessment form.

*All with high, complete SCI.

**Table 2:** Bowel function. Cleveland Constipation Score is listed as the total score as well as the individual 8 items of the score.

	Spinal cord injury, *n* = 21	Chronic idiopathic constipation, *n* = 15	*P* value
Total gastrointestinal transit time, median (range), score 0–6.5	3.6 (1.3–6.4)	2.8 (1.3–6.4)	0.48
Cleveland Constipation Score, median (range), score 0–30	11 (4–16)	15 (9–25)	0.01
Frequency of bowel movements, *n *			
1-2 times per 1-2 days	16	5	<0.000
2 times per week	4	0	
Once per week	1	7	
Less than once per week	0	2	
Less than once per month	0	1	
Difficulty: painful evacuation effort, *n *			
Never	13	3	0.021
Rarely	4	1	
Sometimes	2	3	
Usually	1	4	
Always	1	4	
Completeness: feeling incomplete evacuation, *n *			
Never	7	1	0.28
Rarely	2	1	
Sometimes	4	2	
Usually	2	3	
Always	6	8	
Pain: abdominal pain, *n *			
Never	7	2	0.41
Rarely	3	1	
Sometimes	4	2	
Usually	5	6	
Always	2	4	
Time: minutes in lavatory per attempt, *n *			
Less than 5	1	2	0.69
5–10	6	4	
10–20	3	4	
20–30	6	2	
More than 30	5	3	
Assistance: type of assistance			
Without assistance	3	3	0.001
Oral laxatives	3	10	
Digital assistance or enema	15	2	
Failure: unsuccessful attempts for evacuation per 24 hours			
Never	11	11	0.39
1–3	9	4	
3–6	0	0	
6–9	0	0	
More than 9	1	0	
History: duration of constipation (years)			
0	3	1	0.26
1–5	9	4	
5–10	4	2	
10–20	1	5	
More than 20	4	3	

**Table 3:** Abdominal pain characteristics. Data obtained from our 18-item questionnaire.

	Spinal cord injury, *n* = 21	Chronic idiopathic constipation, *n* = 15	*P* value
Number with pain	17	14	0.38
Number with pain duration ≥5 years, *n* (%)	6/16 (37.5)	8/12 (66.7)	0.40
Intensity, median, range, NRS 0–10			
Pain	4 (0–8)	6.5 (4–10)	0.048
Unpleasantness	5.0 (0–9)	7.0 (3–10)	0.048
Maximal pain	7.0 (0–10)	8.0 (4–10)	0.010
Impact, median, range, NRS 0–10			
Daily functions	5.0 (0–9)	6.0 (3–10)	0.11
Mood	5.0 (0–10)	8.0 (0–10)	0.04
Sleep	0 (0–10)	5.0 (0–9)	0.05
Days with pain last week, median (range)	3.5 (0–7)	7.0 (2–7)	0.27
Pain medication for abdominal pain/discomfort, *n* (%)	4/17 (24%)^a^	3/14 (21%)^b^	1.0
Pain/unpleasantness aggravators, *n* (%)			
Constipation	13/17 (77%)	12/14 (86%)	0.66
Food intake	9/17 (53%)	6/14 (43%)	0.72
Cold weather	5/17 (30%)	1/14 (7%)	0.19

^a^Two patients took paracetamol, one tramadol, and one pregabalin, methadone, and venlafaxine.

^b^One patient took paracetamol, one tramadol, and one did not remember the name of the drug.
